# Supplementary material for: A Novel Combination of Blood Biomarkers and Clinical Stroke Scales Facilitates Detection of Large Vessel Occlusion Ischemic Strokes
Source: Diagnostics (Basel). 2021 Jun 22;11(7):1137. doi: 10.3390/diagnostics11071137 (PMC8306880; doi:10.3390/diagnostics11071137)
Supplement: Supplementary file 1 [file diagnostics-11-01137-s001.zip › diagnostics-1233313-supplementary.pdf]

Supplementary Information

Title: Combination of blood biomarkers and stroke scales improves identification of large vessel occlusions

Authors: Edoardo Gaude[1,*], Barbara Nogueira[1], Marcos Ladreda Mochales[1], Sheila Graham[2], Sarah Smith[2], Lisa Shaw[3], Sara Graziadio[4], Gonzalo Ladreda Mochales[1], Philip Sloan[2], Joshua D. Bernstock[5], Shashank Shekhar[6], Toby I. Gropen[7], Christopher I. Price[3]

Supplementary Tables

Supplementary Table S1.

	All	LVO	NonLVO	P-value
n	128	23	105	
**D-dimer (µg/mL)**	**0.5 (0.66)**	**1.3 (2.0)**	**0.4 (0.4)**	**< 0.001 *****
OPN (ng/mL)	1.3 (1.2)	1.7 (1.1)	1.2 (1.1)	0.02 *
**OPG (pg/mL)**	**100 (63)**	**125 (60)**	**96 (54)**	**0.01 ***
vWF (µg/mL)	12.4 (6.9)	12.9 (7.4)	12 (7.3)	0.16
**ADAMTS13 (ng/mL)**	**1000 (303)**	**1074 (297)**	**985 (306)**	**0.47**
	**All**	**Haemorrhagic**	**Ischaemic / Non-stroke**	**P-value**
GFAP (pg/mL)	188 (952)	1043 (2581)	66 (127)	0.03 *

Plasma concentrations of blood biomarkers measured in all patients, LVO, and non-LVO patients. Median values are reported with IQR values shown in brackets. Wilcoxon-Mann Whitney p-values are shown.

Supplementary Table S2.

	All	LVO	Non-LVO	P-value
N	122	23	99	
C_STAT	0 (1)	1 (2)	0 (0)	< 0.001
EMSA	3 (4)	5 (1)	2 (3)	< 0.001
FAST	1 (1)	2 (1)	1 (2)	< 0.001
FAST_ED	1 (3)	5 (3)	1 (2)	< 0.001
FAST	1 (1)	2 (1)	1 (2)	< 0.001
RACE	1 (3)	7 (6)	0 (2)	< 0.001

Median values of stroke severity scales obtained for all patients, LVO, and non-LVO patients. IQR values shown in brackets. Wilcoxon-Mann Whitney was used to calculate p-values.

Supplementary Table S3.

Model	Parameters	Beta	CI	OR (95% CI)	Pvalue
C-STAT	C-STAT	2.32	0.47 to 1.28	2.35 (1.60 to 3.61)	<0.001
C-STAT + biomarkers	C-STAT	3.02	0.6 to 1.73	3.03 (1.82 to 5.67)	<0.001
	**D-dimer**	**3.26**	**0.75 to 2.16**	**16.56 (4.46 to 75.93)**	**<0.001**
	GFAP	-2.48	-1.79 to -0.36	0.15 (0.03 to 0.48)	<0.01
EMSA	EMSA	4.47	0.56 to 1.4	2.54 (1.74 to 4.07)	<0.001
EMSA + biomarkers	EMSA	5.7	0.68 to 1.86	3.29 (1.98 to 6.4)	<0.001
	**D-dimer**	**3.38**	**0.74 to 2.32**	**18.4 (4.44 to 103.73)**	**<0.001**
	GFAP	-3.15	-2.32 to -0.41	0.09 (0.01 to 0.44)	0.01
FAST	FAST	3.79	0.91 to 2.27	4.61 (2.48 to 9.71)	<0.001
FAST + biomarkers	FAST	4.85	1.14 to 3	7.07 (3.12 to 20.06)	<0.001
	**D-dimer**	**3.47**	**0.79 to 2.36**	**19.79 (4.83 to 111.98)**	**<0.001**
	GFAP	-2.77	-2.08 to -0.35	0.12 (0.02 to 0.5)	0.01
FAST-ED	FAST-ED	4.14	0.48 to 1.03	2.07 (1.62 to 2.8)	<0.001
FAST-ED + biomarkers	FAST-ED	6.3	0.7 to 1.67	3.03 (2.02 to 5.3)	<0.001
	**D-dimer**	**3.66**	**0.7 to 2.67**	**23.43 (4.06 to 209.78)**	**0.001**
	GFAP	-3.95	-2.79 to -0.73	0.05 (0 to 0.23)	<0.01
RACE	RACE	3.9	0.36 to 0.74	1.7 (1.43 to 2.1)	<0.001
RACE + biomarkers	RACE	5.01	0.43 to 1.05	1.98 (1.53 to 2.86)	<0.001
	**D-dimer**	**3.5**	**0.71 to 2.47**	**20.38 (4.14 to 138.41)**	**<0.001**
	GFAP	-3.57	-2.91 to -0.43	0.06 (0 to 0.43)	0.03

Univariate and multivariable logistic regression models for LVO prediction.

The p-values indicate statistical significance of the model covariate.

Supplementary Table S4.

	Hemorrhagic	Ischemic LVO	Ischemic SVO	Mimics	TIA
Reference Standard	16	23	42	26	15
**DDimer+GFAP**	**1**	**13**	**5**	**1**	**2**
C-STAT	3	9	1	3	0
**C-STAT+DDimer+GFAP**	**1**	**17**	**3**	**2**	**1**
EMSA	1	4	1	0	0
**EMSA+DDimer+GFAP**	**1**	**20**	**4**	**1**	**0**
FAST	2	5	1	0	1
**FAST+DDimer+GFAP**	**2**	**19**	**4**	**1**	**2**
FAST-ED	4	16	0	3	0
**FAST-ED+DDimer+GFAP**	**1**	**21**	**0**	**3**	**0**
RACE	5	16	0	1	0
**RACE+DDimer+GFAP**	**3**	**20**	**2**	**3**	**0**

Number of patients selected by each model or stroke scale, compared to the reference standard. Number of patients selected are divided into the different subtypes of suspected stroke.

Supplementary Table S5.

Scale	All	LVO	NonLVO	Pvalue
C_STAT	0 (1)	3 (3)	0 (0)	p < 0.001
EMSA	3 (3.5)	5 (1)	2.5 (3)	p < 0.001
FAST	2 (1)	3 (1)	1 (1.75)	p < 0.001
FAST_ED	1 (3)	7 (2)	1 (2)	p < 0.001
RACE	1 (4.5)	9 (1)	1 (2)	p < 0.001

Median values of stroke severity scales obtained for all patients, LVO, and non-LVO patients. IQR values shown in brackets. Wilcoxon-Mann Whitney was used to calculate p-values.

Supplementary Figures

Supplementary Figure S1. Diagnostic categorization.

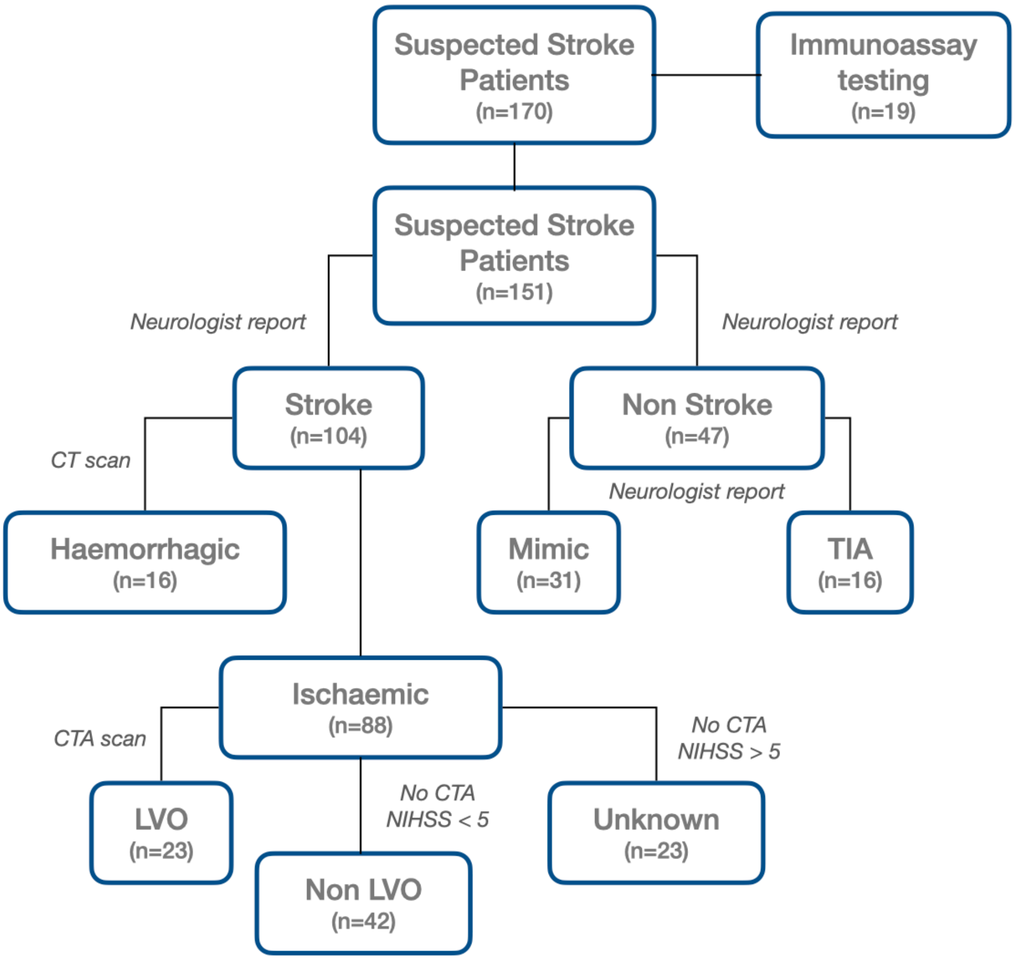

Supplementary Figure S1. Each box represents different types of stroke subtype or suspected stroke. Number of each patient categorised in different subtypes is reported in brackets. Italic text outside each box indicates the methods used to assign diagnosis.

Supplementary Figure S2. Bi-directional stepwise feature selection.

```
Start:  AIC=122.56
Diagnosis2 ~ 1

           Df Deviance    AIC
+ DDimer    1   98.973 102.97
+ OPG       1  116.047 120.05
+ OPN       1  117.372 121.37
<none>         120.555 122.56
+ vWF       1  118.776 122.78
+ GFAP      1  119.000 123.00
+ ADAMTS13  1  120.092 124.09

Step:  AIC=102.97
Diagnosis2 ~ DDimer

           Df Deviance    AIC
+ GFAP      1   94.332 100.33
+ ADAMTS13  1   96.278 102.28
<none>          98.973 102.97
+ OPN       1   96.981 102.98
+ OPG       1   98.486 104.49
+ vWF       1   98.972 104.97
- DDimer    1  120.555 122.56

Step:  AIC=100.33
Diagnosis2 ~ DDimer + GFAP

           Df Deviance    AIC
<none>          94.332 100.33
+ OPN       1   92.340 100.34
+ ADAMTS13  1   92.790 100.79
+ OPG       1   93.871 101.87
+ vWF       1   94.128 102.13
- GFAP      1   98.973 102.97
- DDimer    1  119.000 123.00
```

Supplementary Figure S2. Output of the bi-directional multi-step selection performed on the panel of 6 biomarkers. The steps show how the selection of D-dimer and GFAP was achieved based on AIC values.
